# Validity, reliability, and calibration of the physical activity unit 7 item screener (PAU-7S) at population scale

**DOI:** 10.1186/s12966-021-01169-w

**Published:** 2021-07-17

**Authors:** Helmut Schröder, Isaac Subirana, Julia Wärnberg, María Medrano, Marcela González-Gross, Narcis Gusi, Susana Aznar, Pedro E. Alcaraz, Miguel A. González-Valeiro, Lluis Serra-Majem, Nicolás Terrados, Josep A. Tur, Marta Segú, Clara Homs, Alicia Garcia-Álvarez, Juan C. Benavente-Marín, F. Javier Barón-López, Idoia Labayen, Augusto G. Zapico, Jesús Sánchez-Gómez, Fabio Jiménez-Zazo, Elena Marín-Cascales, Marta Sevilla-Sanchez, Estefanía Herrera-Ramos, Susana Pulgar, María del Mar Bibiloni, Clara Sistac-Sorigué, Santiago F. Gómez

**Affiliations:** 1 Ciber Epidemiology and Public Health (CIBERESP), Instituto de Salud Carlos III, Madrid, Spain; 2 Cardiovascular Risk and Nutrition Research Group, IMIM (Hospital del Mar Medical Research Institute), Barcelona, Spain; 3 Cardiovascular Epidemiology and Genetics Research Group, IMIM Hospital del Mar Medical Research Institute, Barcelona, Spain; 4 Centro de Investigación Biomédica en Red de Enfermedades Cardiovasculares (CIBERCV), Madrid, Spain; 5 School of Health Sciences, Universidad de Málaga-Instituto de investigación biomédica de Málaga, Málaga, Spain; 6 Centro de Investigación Biomédica en Red Fisiopatología de la Obesidad y la Nutrición (CIBEROBN), Institute of Health Carlos III, Madrid, Spain; 7 ELIKOS group, Institute for Innovation & Sustainable Development in Food Chain (IS-FOOD), Instituto de Investigación Sanitaria de Navarra (IDISNA), Public University of Navarra, Navarra, Spain; 8 ImFINE Research Group, Department of Health and Human Performance, Universidad Politécnica de Madrid, Madrid, Spain; 9 Physical Activity and Quality of Life Research Group (AFYCAV), Faculty of Sport Sciences, University of Extremadura, Cáceres, Spain; 10 PAFS Research Group, Faculty of Sports Sciences, University of Castilla-La Mancha, Toledo, Spain; 11 CIBER of Frailty and Healthy Aging (CIBERFES), Madrid, Spain; 12 Research Center for High Performance Sport, Catholic University of Murcia, Murcia, Spain; 13 Faculty of Sport Sciences, UCAM, Catholic University of Murcia, Murcia, Spain; 14 Faculty of Sports Sciences and Physical Education, University of A Coruña, A Coruña, Spain; 15 Research Institute of Biomedical and Health Sciences (IUIBS), University of Las Palmas de Gran Canaria, Las Palmas, Spain; 16 Preventive Medicine Service, Canarian Health Service, Centro Hospitalario Universitario Insular Materno Infantil (CHUIMI), Las Palmas, Spain; 17 Regional Unit of Sports Medicine, Municipal Sports Foundation of Avilés, Asturias, Spain; 18 Research Group of Community Nutrition & Oxidative Stress, University of the Balearic Islands, Palma de Mallorca, Spain; 19 Probitas Foundation, Barcelona, Spain; 20 Gasol Foundation, 08830 Sant Boi de Llobregat, Spain; 21 GRoW, Global Research on Wellbeing, Blanquerna School of Life Sciences, University Ramon Llull, Barcelona, Spain; 22 Faculty of Health Sciences, Universitat Oberta de Catalunya, Barcelona, Spain; 23 Department of Didactics of Language, Arts and Physical Education, Universidad Complutense de Madrid, Madrid, Spain; 24 GREpS, Health Education Research Group, Nursing and Physiotherapy Department, University of Lleida, Lleida, Spain

**Keywords:** Children and adolescents, Self-reported physical activity, Short PAQ, Validation, Accelerometry

## Abstract

**Background:**

Validation of self-reported tools, such as physical activity (PA) questionnaires, is crucial. The aim of this study was to determine test-retest reliability, internal consistency, and the concurrent, construct, and predictive validity of the short semi-quantitative Physical Activity Unit 7 item Screener (PAU-7S), using accelerometry as the reference measurement. The effect of linear calibration on PAU-7S validity was tested.

**Methods:**

A randomized sample of 321 healthy children aged 8–16 years (149 boys, 172 girls) from the nationwide representative PASOS study completed the PAU-7S before and after wearing an accelerometer for at least 7 consecutive days. Weight, height, and waist circumference were measured. Cronbach alpha was calculated for internal consistency. Test-retest reliability was determined by intra-class correlation (ICC). Concurrent validity was assessed by ICC and Spearman correlation coefficient between moderate to vigorous PA (MVPA) derived by the PAU-7S and by accelerometer. Concordance between both methods was analyzed by absolute agreement, weighted kappa, and Bland-Altman statistics. Multiple linear regression models were fitted for construct validity and predictive validity was determined by leave-one-out cross-validation.

**Results:**

The PAU-7S overestimated MVPA by 18%, compared to accelerometers (106.5 ± 77.0 vs 95.2 ± 33.2 min/day, respectively). A Cronbach alpha of 0.76 showed an acceptable internal consistency of the PAU-7S. Test-retest reliability was good (ICC 0.71 *p* < 0.001). Spearman correlation and ICC coefficients of MVPA derived by the PAU-7S and accelerometers increased from 0.31 to 0.62 and 0.20 to 0.62, respectively, after calibration of the PAU-7S. Between-methods concordance improved from a weighted kappa of 0.24 to 0.50 after calibration. A slight reduction in ICC, from 0.62 to 0.60, yielded good predictive validity. Multiple linear regression models showed an inverse association of MVPA with standardized body mass index (β − 0.162; *p* < 0.077) and waist to height ratio (β − 0.010; *p* < 0.014). All validity dimensions were somewhat stronger in boys compared to girls.

**Conclusion:**

The PAU-7S shows a good test-retest reliability and acceptable internal consistency. All dimensions of validity increased from poor/fair to moderate/good after calibration. The PAU-7S is a valid instrument for measuring MVPA in children and adolescents.

**Trial registration:**

**Trial registration number** ISRCTN34251612.

**Supplementary Information:**

The online version contains supplementary material available at 10.1186/s12966-021-01169-w.

## Introduction

Physical activity (PA) is associated with favorable mental and physical health in children and adolescents [1–3]. The World Health Organization (WHO) recommends at least 60 min per day of moderate and vigorous physical activity (MVPA) for children aged 5 to 17 years [4]. This recommendation is shared by most European countries, including Spain [5].

Measurement of daily PA is paramount to identify children not meeting current recommendations and implement intervention programs aimed to promote PA that can engage this at-risk population. However, the measurement of true PA is challenging. Objective methods to measure PA, such as accelerometry, are difficult to implement in large-scale epidemiological studies and time-limited settings due to economic and logistic burdens [6, 7]. Additionally, PA measurement by accelerometers has several limitations making it difficult to compare data between studies [8]. In comparison, the administration of PA questionnaires is a cheaper and more feasible method, albeit less accurate, to meet the challenge of measuring PA in children and adolescents. These questionnaires vary in their design and structure (e.g., recorded periods of PA range from 1 day to 1 year) [7] and have been validated in specific population subgroups, which limits the transferability of results. Additionally, most questionnaires for children and adolescents include qualitative questions, ask for details about PA frequencies, and are generally too complex for use in time-limited settings such as the pediatrician’s daily practice [7]. Several short quantitative PA questionnaires are available but were validated in specific populations [9–12], which limits their use in other populations. Furthermore, these questionnaires are limited for cross-nation comparison of PA*.* Therefore, brief PA questionnaires are needed to readily identify children not meeting the WHO PA recommendations and assist in PA counseling [13]. For this reason we developed the Physical Activity Unit 7-day Screener (PAU-7S), a brief PA questionnaire developed to measure PA in children and adolescents.

PA questionnaires are generally developed specifically for each study population and research aim due to the impact of ethnicity, culture, behavior, and biology on PA (7). Therefore, the validity of PA questionnaires beyond the target study population is limited. Furthermore, measurement error of self-reported data is a concern. The available calibrated questionnaire-derived PA data, although scant, show promising results [11, 14–18].

The aim of the present study was to determine the reliability and the concurrent, construct, and predictive validity of PAU-7S in a randomized, nationally representative subsample of the PASOS study of children and adolescents aged 8 to 16 years. Additionally, we evaluated the effect of linear regression calibration on each of the three dimensions of PAU-7S validity.

## Methods

### Participants

This validation study was performed within the frame of a nationwide representative study of Physical Activity, Sedentarism, lifestyles and Obesity in Spanish youth (PASOS). The methodology of the PASOS study has been described in detail elsewhere [19]. In brief, a representative sample from 22 school groups of 4508 children aged 8–16 years and their parents were invited, of whom 3817 agreed to participate (84.7% response rate).

Of this study population, a randomized sample of 389 (10.2%) children and adolescents was invited to participate in the validation study and 369 (94.9% response rate) agreed to participate. For test-retest reliability analysis, 321 participants completed PAU-7S questionnaires at baseline and after 1 week of wearing an accelerometer. After excluding 17 participants with missing or invalid accelerometer data, 304 participants were included in the validity analysis. Participants did not receive any compensation for their participation*.* The study protocol was approved by the Ethics Committee CEIm Fundació Sant Joan de Déu, Spain (Approval number: PIC-171-18). Parental written informed consent was obtained.

### Development and administration of the PAU-7S

The development of the PAU-7S involved three strategies: (i) review of validated PA questionnaires for children; (ii) consulting PA experts from the IMIM-Hospital del Mar Research Institute; (iii) analysis of PA data of the Thao-POIBC [20] and EnKid [21] studies to identify activities that explain the variability of PA in children and young adolescents.

The resulting 7-day PA questionnaire was designed to measure regular PA in a typical week. Most PA questionnaires commonly used with children focus on this timeframe because they show good weekly recall [22]. The PAU-7S questionnaire design considered the usual opportunities to do PA during the day. The online questionnaire included 6 main questions about the previous week: 1. How many days did you go for a walk? 2. How many days were you engaged in active play during recess time? 3. How many days were you engaged in active play during free time after school or during the weekend? 4. How many days did you have Physical Education (PE) class at school? 5. How many days did you play a team sport? (for example: soccer, basket, handball, hockey, and water polo). 6. How many days did you play individual sports? (for example: track and field, eurythmics, dance-ballet, tennis, judo-karate-taekwondo, roller-skating, swimming). For each question, the answering options were presented as a table showing each day with spaces where the children would mark if they spent (i) less than 30 min on the activity that day; (ii) 30 min to one hour; (iii) one hour to one hour and a half; or (iv) more than one hour and a half. Children had to select an option before progressing through the online system. For the second and fourth questions, which ask about physical activity during school time, response options were only shown from Monday to Friday; question 4 did not include time options because a PE class lasts 45 min in Spain. Additionally, two qualitative items were included i) Are any of these sports aquatic activities? (Yes/No) and ii) Were you sick during the past week or did anything prevent you from doing your usual physical activities? The first item was a sub-question of item 5 (How many days did you play a team sport?) and item 6 (How many days did you play individual sports?) The qualitative questions were not used to calculate MVPA*.*

The PAU-7S was administered the first day the accelerometer was worn and 9 days later, when the accelerometer was taken off by trained personnel. MVPA was calculated based on the sum of all activities, with the exception of walking.

### Physical activity measured by accelerometry

PA was measured by the “Actigraph GT3X+” accelerometer (ActiGraph, Pensacola FL- USA), allocating at least 7 days from April to June 2019 for each of the 22 randomized school groups. Children were asked to wear the accelerometer for at least 1 week except while bathing or swimming. The accelerometers were placed on the wrist for the non-dominant hand with a bracelet. The accelerometer data collection protocol was followed by all field workers. A common training session was carried out to ensure the homogeneity of this procedure. The Troiano et al. method [23] was used to identify the time that accelerometers were not worn: periods of 60 min (or more) of zero values were discarded. Data from the accelerometers were considered valid if the accelerometer was worn for at least 4 days with at least 1 weekend day and for at least 10 h between 8 a.m. and 10 p.m. each day. The sampling period was set to 5 epochs (100 Hz) and the outcome was expressed as minutes per day. Chandler et al. cut-off points [24] were used to translate acceleration counts into minutes per week of sedentary, light, moderate, and vigorous PA.

The Actigraph data were downloaded using the software provided by the manufacturer (version 6.0, Actigraph, Pensacola, Florida) and imported into SPSS v21 (IBM, Chicago, IL) for data processing and screening. R package 4.0.2 accelerator (www.datahunter.es) was used to identify wear-time between 8 a.m. and 10 p.m.

### Anthropometric variables

Weight, height, and waist circumferences (WC) measurements were taken by trained personnel, with the children in light clothing, without shoes. The measurements were performed using an electronic SECA 899 scale (recorded to the nearest 100 g), a portable SECA 217 stadiometer (to the nearest 1 mm), and a flexible, non-stretch SECA 201 metric tape (to the nearest 1 mm), respectively. WC was measured in the narrowest zone between the lower costal rib and iliac crest, in the supine decubitus and horizontal positions. BMI z-score was computed using age and sex-specific reference values from the WHO [25]. Waist to-height ratio (WHtR) was calculated.

### Data collection procedure

Following anthropometric and initial weight, height, and WC measurements, participants completed the first PAU-7S during a group session in the computer room at school (1st PAU-7S). Upon questionnaire completion, they received an accelerometer and verbal instructions on its use. Nine days later, participants again completed the questionnaire (2nd PAU-7S).

### Statistical analysis

Participant characteristics were described as mean, standard deviation (SD), and median (inter-quartile range), as appropriate. Distribution of continuous variables between boys and girls were compared using the Student t test for normally distributed variables or Mann-Whitney U test otherwise. Proportion comparisons for categorical variables were assessed using chi-square test. Non-normally distributed variables were log-transformed to achieve normality.

Internal consistency of the PAU-7S questionnaire was tested by Cronbach alpha. Test-retest reliability between PAU-7S-derived basal and 1-week MVPA data was assessed by intra-class correlation coefficients (ICC).

The relative validity of the PAU-7S was assessed by Pearson correlation coefficients comparing MVPA derived by the PAU-7S (test method) to that shown by the accelerometers (criterion standard for validity). Pearson correlation coefficients were classified as follows: > 0.8, very good; 0.61–0.80, good; 0.41–0.60, moderate; 0.21–0.40, fair; and < =0.20, weak [26]. Although the two measurements might be highly correlated, substantial differences between them could exist across the range of values; therefore, we determined absolute agreement between the two measurements by cross-classification and the kappa statistic of tercile distribution of MVPA for both measurements. Concordance between the PAU-7S measurements of MVPA was assessed by kappa values as follows: > 0.8, almost perfect agreement; 0.61–0.80, substantial agreement; 0.41–0.60, moderate agreement; 0.21–0.40, fair agreement; and < =0.20, slight agreement (24).

We further assessed agreement between the two measurements using the original Bland–Altman method [27] and a modified version published by Ludbrook [28]. Both methods calculate the mean of differences between the two measurements and regress it against the mean obtained with each measurement. The method by Ludbrook assumes a possible bias as a function of the mean of each participant and computes the confidence limits accordingly. A mean proportional agreement of 100% between measurements would signify complete agreement; a mean difference of 0 would show complete disagreement between the methods. In addition, we analyzed possible variations in the level of agreement between methods to assess proportional bias. For this purpose, we fitted linear regression models, with the mean instrument differences of MVPA derived by the PAU-7S and accelerometers (MVPA_PAU-7S – MVPA-accelerometers)) as the dependent variable and the mean score of both ((MVPA_PAU-7S + MVPA_accelerometer) / 2)) as the independent variable.

Energy balance is the ratio between energy intake and energy expenditure. Energy expenditure increases with PA [29, 30], which might compensate for excessive energy and its effect on weight gain. Therefore, we hypothesized that a valid construct of the PAU-7S would be inversely associated with body mass index (BMI) and WC. Multiple linear regression models adjusted for sex and age, with anthropometric variables as the outcome and MVPA derived by the PAU-7S as the exposure, were fitted to test construct validity of the PAU-7S. All models were tested for multicollinearity.

To reduce measurement error of the PAU-7S, we performed linear calibration according to Rosner et al. [31]. MVPA derived by accelerometers was regressed against MVPA obtained by the PAU-7S with sex, age, and weight as co-variables. The final calibrated model (MVPA_PAU-7S_calibrated) was as follows: MVPA_PAU-7S_calibrated =183.788 (intercept) + (− 6.374*age) + (1.437*sex) + (0.080* MVPA_PAU-7S) + (− 0.436*weight).

The predictive capacity of the calibration equation was assessed by leave-one-out cross-validation. This iterative procedure predicts the response value of each individual from the model fitted by the rest of the sample. The classification system of interaction between the PAU-7S-derived MVPA, sex, and age was tested.

The Statistical Package for the Social Sciences statistical software package version 21.0 (SPSS Inc., Chicago, IL, USA) was used for all statistical analyses with the exception of leave-one-out cross-validation. This analysis was performed using R package 4.0.2. Differences were considered significant if *p* < 0.05.

## Results

Characteristics of the study population are reported in Table 1. Girls were slightly older, with a higher BMI, compared to boys. At baseline, boys reported higher total PA and MVPA and more minutes spent in team sports and active play outside of school, compared to girls. There was no significant interaction between MVPA derived by the PAU-7S, sex, and age. The comparison of the general characteristics between participants of the validation study (*n* = 323) and those of the remaining PASOS cohort (*n* = 3496) revealed no significant differences with the exception of age. Participants in the validation study were somewhat younger (12.3 ± 2.2 years) than the remaining participants of the PASOS cohort (12.6 ± 2.4) **(Supplementary Table** **1****)**. No significant difference between the sample with accelerometer data (*n* = 304) and the sample for reliability analysis (*n* = 321) was found.

**Table 1:** Characteristics of the study population^a^

	All	Boys	Girls	*p*^b^
	(n = 321)	(*n* = 149)	(*n* = 172)	
Age (years)	12.3 (2.21)	12.0 (2.24)	12.5 (2.15)	0.017
Weight (kg)	47.6 (14.6)	46.4 (14.4)	48.6 (14.8)	0.174
Height (cm)	1.52 (0.13)	1.52 (0.15)	1.52 (0.11)	0.917
BMI (kg/m^2^)	20.2 (4.03)	19.7 (3.46)	20.6 (4.42)	0.031
BMI z score	0.57 (1.25)	0.61 (1.27)	0.53 (1.24)	0.589
Waist circumference (cm)	70.0 (10.6)	70.2 (10.5)	69.8 (10.7)	0.700
WHtR (cm/cm)	0.46 (0.06)	0.46 (0.06)	0.46 (0.06)	0.494
*1st PAU-7S questionnaire*				
Total physical activity (min/d)	167 (92.8)	182 (93.1)	154 (90.8)	0.007
MVPA (min/d)^3^	120 (76.6)	134 (77.5)	108 (73.8)	0.002
Walking (min/d)	40.7 [23.6;66.4]	45.0 [27.9;66.4]	40.7 [23.6;66.4]	0.511
Schoolyard active play (min/d)^c^	10.7 [10.7;32.1]	10.7 [10.7;32.1]	10.7 [8.04;23.6]	0.067
Non-school active play (min/d)^d^	38.6 [17.1;60.0]	45.0 [21.4;68.6]	32.1 [15.0;53.6]	0.017
Physical education (min/d)	4.29 [4.29;4.29]	4.29 [4.29;4.29]	4.29 [4.29;4.29]	0.133
Team sport (min/d)	27.9 [4.29;51.4]	34.3 [12.9;55.7]	15.0 [0.00;45.0]	< 0.001
Individual sport (min/d)	15.0 [0.00;40.7]	12.9 [0.00;45.0]	16.1 [2.14;36.4]	0.707
*2nd PAU-7S questionnaire*				
Total physical activity (min/d)	151 (95.1)	159 (96.4)	144 (93.6)	0.579
MVPA (min/d)	107 (78.2)	114 (79.2)	101 (77.1)	0.135
Walking (min/d)	40.7 [23.6;62.1]	45.0 [23.6;62.1]	36.4 [23.6;57.9]	0.579
Schoolyard active play (min/d)^c^	10.7 [6.43;32.1]	10.7 [10.7;32.1]	10.7 [0.00;28.9]	0.049
Non-school active play (min/d)^d^	32.1 [15.0;57.9]	32.1 [15.0;60.0]	32.1 [12.9;54.1]	0.333
Physical education (min/d)	4.29 [2.14;4.29]	4.29 [4.29;4.29]	4.29 [2.14;4.29]	0.009
Team sport (min/d)	15.0 [0.00;42.9]	25.7 [0.00;45.0]	12.9 [0.00;36.4]	0.039
Individual sport (min/d)	12.9 [0.00;36.4]	10.7 [0.00;32.1]	15.0 [0.00;39.1]	0.180

BMI: Body mass index; MVPA: Moderate to vigorous physical activity; WHtR: Waist to height ratio

^a^Variables are expressed as mean (SD) or median [interquartile range] for normal and non-normal continuous variables, respectively

^b^Gender differences were analyzed by Student t test for independent variables

^c^Physical activity during school recess

^d^Physical activity during free time outside school

The PAU-7S showed good test-retest repeatability for total PA and MVPA in both boys and girls **(**Table 2**)**. The repeatability of each PA activity ranged from moderate to good.

**Table 2:** Test-retest repeatability^a^ of physical activity (PA) recorded by PAU-7S at baseline and after 1 week^b^

	All	Boys	Girls
	(n = 321)	(n = 149)	(n = 172)
Total PA (min/d)	0.73	0.74	0.72
Moderate to vigorous PA (min/day)^c^	0.71	0.71	0.70
Walking (min/d)^d^	0.69	0.70	0.68
Schoolyard active play (min/d)^d,e^	0.47	0.48	0.45
Physical education (min/d)^d^	0.77	0.74	0.78
Non-school active play (min/d)^d,f^	0.61	0.60	0.62
Team sport (min/d)^d^	0.52	0.55	0.47
Individual sport (min/d)^d^	0.68	0.61	0.73

^a^ Intra-class correlation coefficient

^b^ *p* < 0.001 for all correlations

^c^ Walking was excluded

^d^ Log-transformed before analysis

^e^ Physical activity during school recess

^f^ Physical activity during free time outside school

**Fig. 1:**
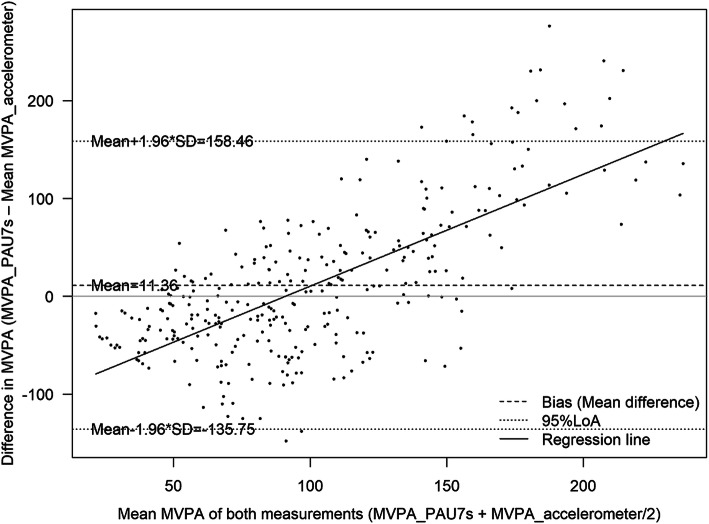
Bland-Altman plot for the agreement of moderate-to-vigorous physical activity (MVPA) derived from the un-calibrated Physical Activity Unit 7 item Screener (PAU-7S) and the accelerometer (N = 301)

The Cronbach alpha of 0.76 indicated acceptable internal consistency of the PAU-7S, with a slightly better result in girls than in boys **(**Table 3**)**. The noncalibrated PAU-7S significantly overestimated MVPA (by 18%) compared to the criterion standard for validity**;** furthermore, this discrepancy significantly increased (β coefficient 0.428 (0.379;0.478**)** with higher levels of MVPA **(**Table 3 and Fig. 1**)**. Overestimation of MVPA was somewhat greater in boys than in girls **(**Table 3**)** and in adolescents compared to children **(Supplementary Table** **2****)**. Table 3 also shows Pearson correlation coefficients between methods, indicating the capacity of the PAU-7S to rank levels of MVPA in children. Pearson coefficients between MVPA derived by the PAU-7S and by accelerometers revealed a fair concurrent validity of the PAU-7S overall as well as separately for boys and girls and for children and adolescents **(Supplementary Table** **2**.

**Table 3:** Correlation coefficients and between-method agreement of moderate to vigorous physical activity (MVPA) measurements derived by the Physical Activity Unit 7 item screener, noncalibrated and calibrated, and the reference method (accelerometer)

	All (n = 304)	Boys (*n* = 141)	Girls (*n* = 163)
*Accelerometer*			
MVPA, min/d (SD)	95.2 (33.2)	97.2 (33.8)	93.4 (32.7)
*PAU-7S, noncalibrated*			
MVPA, min/d (SD)	106.5 (77.0)	111.0 (77.2)	102.7 (76.9)
Between-method difference, min/d (95% CI)^a^	11.4 (2.9;19.8)	13.8 (1.5;26.0)	9.3 (−2.5;21.1)
Proportional agreement, %; (95% CI)^b^	118 (107;128)	120 (106;134)	116 (101;131)
Cronbach alpha	0.76	0.73	0.78
Regression coefficient^c^	0.428 (0.379;0.478)	0.444 (0.367;0.520)	0.414 (0.349;0.479)
Spearman correlation coefficient	0.31	0.33	0.29
Intra-class correlation coefficient	0.20	0.24	0.16
Absolute agreement, %^d^	46.7	48.2	45.4
Gross misclassification, %^e^	14.1	14.9	13.5
Kappa^f^	0.24	0.24	0.24
*PAU-7S, calibrated*	95.2 (33.2)	97.2 (33.8)	93.4 (32.7)
Between-method difference, min/d (95% CI)^a^	0 (−3.3;3.3)	1.3 (−3.4;5.6)	−1.09 (−5.6;3.5)
Proportional agreement, %; (95% CI)^b^	105 (101;109)	106 (100;112)	104 (99;110)
Regression coefficient^c^	1.9*10^−6^ (−0.116;0.116)	0.007 (−0.177;0.192)	−0.012 (−0.160;0.137)
Spearman correlation coefficient	0.62	0.66	0.57
Intra-class correlation coefficient	0.62	0.66	0.57
Absolute agreement, %^d^	59.2	65.2	54.0
Gross misclassification, %^e^	3.2	1.8	3.7
Kappa^f^	0.50	0.57	0.44

CI: confidence interval; MVPA: moderate to vigorous physical activity; PAU-7S: Physical Activity Unit 7-item screener; SD: standard deviation

^a^Calculated as: MVPA_PAU-7S – MVPA_accelerometer

^b^Calculated as: [MVPA_accelerometer/MVPA-PAU-7S] * 100

^c^Regression coefficients (β) between mean of the MVPA and mean differences (independent variable) between MVPA obtained by the PAU-7S and by accelerometers

^d^Correctly classified terciles of MVPA derived by the PAU-7S and by accelerometers

^e^Opposite terciles of MVPA derived by the PAU-7S and by accelerometers

^f^Weighted kappa between terciles of MVPA derived by the PAU-7S and accelerometers

The absolute agreement of the PAU-7S as measured by correct cross-classification of tercile distribution of MVPA by the two methods was 46.7% for the entire population; somewhat higher values were observed for boys (48.2%) compared to girls (45.2%) **(**Table 3**).** Additional kappa statistics, which account for agreement by chance, showed fair concordance between the two methods, with the identical kappa value in boys and girls (k = 0.24) **(**Table 3**).**

Multiple linear regression models adjusted for sex and age revealed an inverse association of MVPA derived by PAU-7S with WHtR (*p* = 0.014) and standardized BMI (zBMI) (*p* = 0.077), as shown in Table 4. Calibration models showed a significant collinearity. Therefore, age and sex were excluded from the final model.

**Table 4:** Multiple linear regression^a^ of the association between PAU-7S-derived moderate to vigorous physical activity and anthropometric markers of body fat

	Β coefficient^b^	95% CI	*p*
PAU-7S, *non-calibrated*			
zBMI (kg/m^2^)	−0.162	− 0.345;0.018	0.077
WHtR (cm/cm)	−0.010	−0.019;-0.001	0.014
PAU-7S, *calibrated*			
zBMI (kg/m^2^)	−0.216	−0.638;0.200	0.316
WHtR (cm/cm)	−0.025	−0.044;-0.006	0.009

CI: Confidence interval; PAU-7S: physical activity unit 7-item screener; zBMI: standardized body mass index; WHtR: waist to height ratio

^a^Adjusted for sex and age

^b^Per 100 min of MVPA per day

Concurrent validity considerably improved after linear calibration of the PAU-7S **(**Table 3**)**. Pearson and ICC coefficients increased to 0.62 for both of these dimensions, with similar results in both boys and girls. The concordance between methods as measured by absolute agreement and kappa statistic improved to 59.2% and 0.50, respectively. The regression coefficients of the MVPA association with zBMI and WHtR slightly increased after calibration of the PAU-7S **(**Table 4**)**.

**Fig. 2:**
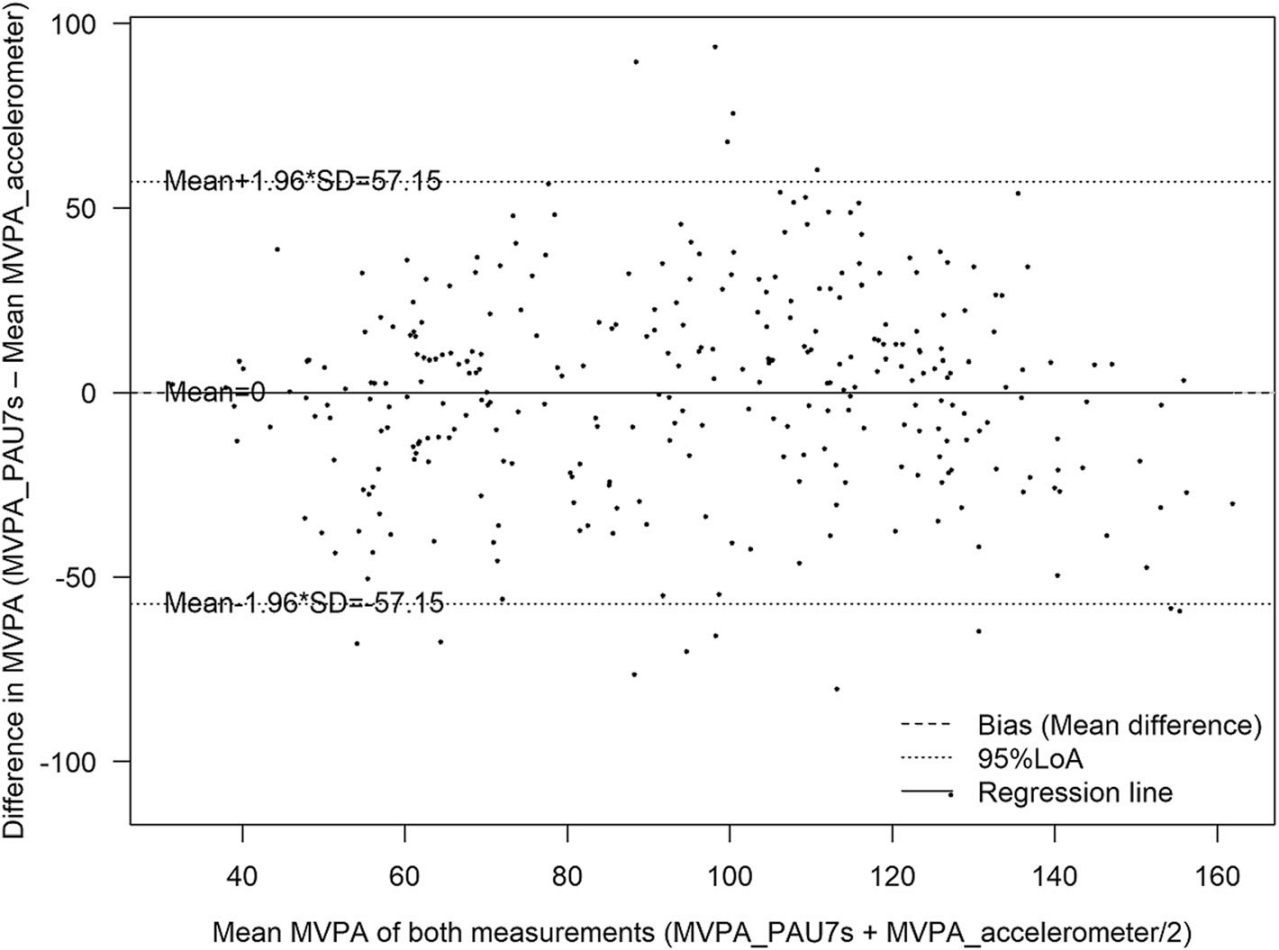
Bland-Altman plot for the agreement of moderate-to-vigorous physical activity (MVPA) derived from the calibrated Physical Activity Unit 7 item Screener (PAU-7S) and the accelerometer (*N* = 301)

**Fig. 3:**
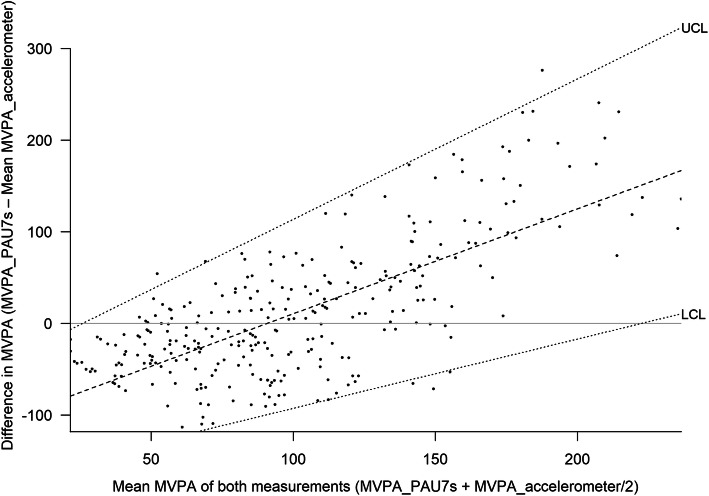
Bland-Altman plot according to Ludbrook (28) for the agreement of moderate-to-vigorous physical activity (MVPA) derived from the un-calibrated Physical Activity Unit 7 item Screener (PAU-7S) and the accelerometer (N = 301)

**Fig. 4:**
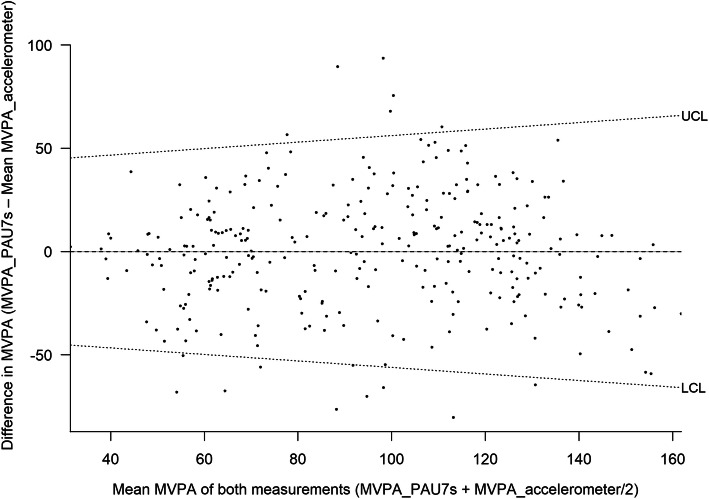
Bland-Altman plot according to Ludbrook (28) for the agreement of moderate-to-vigorous physical activity (MVPA) derived from the calibrated Physical Activity Unit 7 item Screener (PAU-7S) and the accelerometer (N = 301)

The mean agreement between MVPA reported by accelerometers (criterion standard for validity) is shown in Figs. 2 and 3 and the non-calibrated and calibrated PAU-7S data in Figs. 4 and 5. The non-calibrated PAU7s shows a significantly overestimation of MVPA and a significant proportional bias. The predicted difference in MVPA between the PAU7s and accelerometers increased by 0.428 (*p* < 0–05) and 1.9 * 10^− 6^ (*p* > 0.05) min/d per each minute of the mean MVPA obtained by both methods, for the non-calibrated and calibrated PAU7s, respectively. This fact indicates that the calibration of the questionnaire eliminated the proportional bias.

Cross-validation analysis of the predictive validity of the PAU-7S showed a slight reduction of Pearson and ICC coefficients from the original validation sample to the test sample from 0.62 (95% CI 0.54;0.67) to 0.60 (95% CI 0.55;0.0.69) and from 0.62 (95% CI 0.55;0.0.69) to 0.60 (95% CI 0.53;0.67).

## Discussion

The PAU-7S showed a good test-retest reliability and acceptable internal consistency. The questionnaire fairly ranked children according to levels of MVPA, with slightly better results in boys and adolescents compared to girls. Most importantly, although the noncalibrated PAU-7S overestimated MVPA, especially at higher levels of activity, linear calibration meaningfully increased the concurrent and construct validity of the questionnaire.

In general, the ability of PA questionnaires to adequately measure PA in children and adolescents is modest at best [32, 33]. Although objective measurement of PA by accelerometry is an option, it is not always feasible due to economic and logistical burdens, and accelerometer-derived data lack information on context and type of activity (7).

The consistency of participant responses across the items of the PAU-7S was determined by Cronbach alpha. In general, all the questionnaire items are supposed to reflect the same underlying construct, and therefore should be correlated with each others [34]. The internal consistency of the PAU-7S is within an acceptable range (Cronbach alpha =0.76) [35] and comparable to that of other PA questionnaires used in youth [36, 37]. Test-retest reliability for total PA and MVPA derived by the PAU-7S was good overall and not meaningfully different between boys and girls. The observed ICC for MVPA was lower than that of the Spanish adaptation of the Physical Activity Questionnaire for Children (PAQ-C) [37]. However, the nearly perfect test-retest repeatability (ICC 0.96) of the PAQ-C is likely due to the short timeframe for the second administration of the questionnaire, within 6 h of baseline. A recently published work in 712 Spanish children and adolescents showed a good test-retest reliability for the Spanish version of the Youth Activity Profile (YAP) questionnaire [38]. The YAP questionnaire was administered 2 weeks apart, yielding an ICC of 0.66 and 0.72 in children and adolescents, respectively. The ICC of the test-retest reliability of the PAU-7S after 9 days is comparable to that found by Martínez-Gómez and colleagues for the Spanish version of the PAQ-A (for adolescents), administered with a 1-week retest timeframe [36]. Furthermore, the PAU-7S showed considerably better test-retest reliability compared to the short form (7 items) of the International Physical Activity Questionnaire (IPAQ; IPAQ-SF) administered in Norwegian adolescents [39].

In the noncalibrated PAU-7S data, MVPA was overestimated by 11.4 min per day in comparison to accelerometer-derived MVPA. This is considerably lower than the IPAQ-A overestimation by 39.8 min of MVPA in Spanish adolescents [40]. Furthermore, the IPAQ-SF overestimates MVPA in a range from 36 to 173% in five studies, whereas one study reports an underestimation of 28% [41].

The Spearman correlation of 0.31 for concurrent validity in the present study falls within the range reported for most PA questionnaires for youth [22, 33]; we would note that few PA questionnaires for children and adolescents have been validated in the Spanish population (33,34,37,39,40). However, the information yield by most of these questionnaires is limited due to the few PA domains included, as several ask only one question about out-of-school sport activities, or total PA during the day, or yield data for a qualitative comparison of PA level with that of other children (37). The concurrent validity of the PAQ-A and PAQ-C administered in Spanish adolescents and children, respectively, ranged from fair to moderate (31,34,37). The highest concurrent validity (r = 0.54) was found for the adapted version of the Assessment of Physical Activity Levels Questionnaire (APALQ) among Spanish adolescents [10]. The Patient-Centered Assessment and Counselling for Exercise Plus Nutrition (PACE+) is a two-item PA questionnaire developed to estimate compliance with the PA-Guidelines for youth (1). The 60-min MVPA composite of the PACE+ showed fair to moderate validity for girls and boys, respectively, when compared to accelerometer-derived MVPA.

In most of the previously published studies, determination of the validity of the questionnaires used was limited to the assessment of concurrent validity by Spearman or Pearson correlation coefficients. This analysis can yield insights into the capacity of a questionnaire to rank children according to PA levels (e.g., low, medium, or high) but cannot assess absolute agreement between the PA questionnaire and the criterion standard of validity. In the present study, we found a somewhat lower ICC of MVPA between methods compared to that reported by Martín-Bello and colleagues [40] for the IPAQ-A. The absolute agreement of 46.7% correctly classified adolescents according to tercile classification of MVPA by our questionnaire and by accelerometer-derived MVPA, in addition to a kappa value of 0.24; this indicates a fair agreement for the non-calibrated PAU-7S. Furthermore, the Bland-Altman plot revealed a significant bias across the range of MVPA estimates between questionnaire and accelerometer data, showing an increasing measurement error at higher levels of MVPA on the PAU-7S.

Relatively few studies have addressed this issue for self-reported PA assessment in children (11–16). In our study, Pearson and Spearman correlation coefficients increased to 0.63 and 0.62, respectively, after calibration of PAU-7S-derived MVPA estimates. Absolute agreement between methods was moderate (kappa = 0.50) for calibrated estimates of MVPA. Furthermore, the beta coefficient of the association between MVPA derived by the PAU-7S and the WHtR and zBMI score increased from − 0.010 to − 2.46 and from − 0.162 to − 5.850, respectively. This finding indicates a considerable improvement of the construct validity of the PAU-7S after calibration. Finally, the predictive validity of the calibrated PAU-7S data was good, according to cross-validation with an independent internal sample. These results clearly show that linear calibration of MVPA derived by the PAU-7S strongly improved all validity dimensions tested. This finding is in line with the scarce evidence from other PA questionnaire calibration studies in children (11,12,14–16). Saint-Maurice and colleagues found a reasonable ability of the PAQ-C and PAQ-A calibration algorithm to estimate group-level estimates of accelerometer activity (15). Similar results were reported for the YAP questionnaires (14), the Global Physical Activity Questionnaire (12), and the Previous Day Physical Activity Recall (11) calibration algorithm.

The main strength of the present study is the population-based design, which permits generalization of the results to other Spanish populations of children aged 8–16 years. The inclusion of multiple dimensions of validity –concurrent, construct, and predictive– also can be considered a strength of this study. The short length of the PAU-7S makes it ideal for time-limited settings such as primary care centres and for large epidemiological studies or those that attempt to evaluate a long list of indicators. In addition, it will be useful for monitoring national PA data and for comparison with other countries. Finally, these results provide evidence that calibration can improve the validity of PA questionnaires for children and adolescents. This study also has two limitations that must be noted. Accelerometers are not sensitive to PA such as cycling or aquatics (7), and measurement error of self-reported data is an inherent limitation of questionnaires. Furthermore, the PAU-7S includes only 6 general questions about physical activities, which allows only a rough overview of the PA pattern and of METs spent in physical activities. For example, METs of sport activities are specific for each sport but the PAU-7S asks globally for time spent in team and individual sport activities. Therefore, the PAU-7S contribution to calculating the corresponding METs of these activities is limited. Hence, it should not be used when a more exact estimation of METs is the purpose of the study. Furthermore, a single administration of the PAU-7S will not accurately reveal seasonal variation in physical activities.

## Conclusion

The PAU-7S is a valid instrument for the measurement of physical activity in Spanish children and adolescents aged 8 to 16 years. The questionnaire is an adequate instrument for a general estimation of PA, especially in time-limited settings such as primary care and in epidemiological surveys with a large sample size or with many measures of other health indicators where the administration of accelerometers is not feasible. The calibration of this questionnaire meaningfully decreased measurement error and thereby increased its validity. Further studies are needed to shed light on the external validity of the PAU-7S.

## Supplementary Information

**Additional file 1 Supplementary Table 1.** Characteristics of the validation study participants and the remaining participants of the population-based PASOS cohort.

**Additional file 2 Supplementary Table 2.** Correlation coefficients and between-method agreement of moderate to vigorous physical activity measurements derived by the Physical Activity Unit 7-item screener, noncalibrated and calibrated, and the reference method (accelerometer), stratified by age.

## Data Availability

The dataset from the current analysis is not publicly but is available from the corresponding author upon reasonable request.
