# Supplementary material for: Validity, reliability, and calibration of the physical activity unit 7 item screener (PAU-7S) at population scale
Source: Int J Behav Nutr Phys Act. 2021 Jul 17;18:98. doi: 10.1186/s12966-021-01169-w (PMC8285783; doi:10.1186/s12966-021-01169-w)
Supplement: Supplementary file 1 — Additional file 1 Supplementary Table 1. Characteristics of the validation study participants and the remaining participants of the population-based PASOS cohort. [file 12966_2021_1169_MOESM1_ESM.docx]

**Supplementary Table 1.** Characteristics of the validation study participants and the remaining participants of the population-based PASOS cohort.

|  | Validation study  (n=321) | Validation study  (n=304) | PASOS  (n=3496) | *p^a^* | *p^b^* |
| --- | --- | --- | --- | --- | --- |
| Sex. male | 46.3 | 46.4 | 48.1 | 0-993 | 0.581 |
| Age, years | 12.3 (2.2) | 12.3 (2.2) | 12.6 (2.4) | 0.804 | 0.037 |
| BMI, kg/m | 20.2 (4.0) | 20.2 (4.0) | 20.3 (4.0) | 0.989 | 0.500 |
| Waist circumference, cm | 70.0 (10.6) | 70.0 (10.4) | 70.7 (10.9) | 0.987 | 0.222 |
| Paternal education^c^ | 33.6 |  | 30.5 |  | 0.321 |
| MVPA, min/d | 120.1 (76.6) |  | 125.7 (78.3) |  | 0.220 |
| Diet quality, unit^d^ | 6.6 (2.4) | 6.6 (2.5) | 6.8 (2.5) | 0.871 | 0.187 |

Variables are expressed as mean (standard deviation) or proportion. BMI=body mass index; MVPA= Moderate to Vigorous Physical Activity; PASOS= Physical Activity, Sedentarism, and Obesity in Spanish youth.

^a^  Validation study n=321 versus validation study n=304; *p* for differences between samples was performed with Student t and chi-square test for continuous and categorical variables, respectively.

^b^ Validation study n=321 versus PASOS n=3496; *p* for differences see ^a^ .

^c^ University degree.

^d^ Adherence to the Mediterranean diet by the KIDMED index.
