# Supplementary material for: Validity, reliability, and calibration of the physical activity unit 7 item screener (PAU-7S) at population scale
Source: Int J Behav Nutr Phys Act. 2021 Jul 17;18:98. doi: 10.1186/s12966-021-01169-w (PMC8285783; doi:10.1186/s12966-021-01169-w)
Supplement: Supplementary file 2 — Additional file 2 Supplementary Table 2. Correlation coefficients and between-method agreement of moderate to vigorous physical activity measurements derived by the Physical Activity Unit 7-item screener, noncalibrated and calibrated, and the reference method (accelerometer), stratified by age. [file 12966_2021_1169_MOESM2_ESM.docx]

**Supplementary table 2.** Correlation coefficients and between-method agreement of moderate to vigorous physical activity measurements derived by the Physical Activity Unit 7-item screener, noncalibrated and calibrated, and the reference method (accelerometer), stratified by age.

|  | Children (n=150) | Adolescents (n=154) |
| --- | --- | --- |
| *Accelerometer, reference method* |  |  |
| MVPA, min/d (SD*)* | 113.1 (27.6) | 77.7 (28.6) |
| *PAU-7S, noncalibrated* |  |  |
| MVPA, min/d (SD) | 118.0 (7.2) | 95.4 (78.3) |
| Between-method difference, min/d (95% CI)^a^ | 4.9 (-7.3;17.1) | 17.6 (5.8;29.5) |
| Proportional agreement, %; (95% CI)^b^ | 109 (97;143) | 127 (110;143) |
| Cronbach alpha | 0.75 | 0.77 |
| Regression coefficient^c^ | 0.417 (0.359;0.475) | 0.478 (0.416;0.540) |
| Spearman correlation coefficient | 0.12 | 0.35 |
| Intra-class correlation coefficient | 0.09 | 0.24 |
| Absolute agreement, %^d^ | 46.7 | 46.8 |
| Gross misclassification, %^e^ | 15.3 | 13.3 |
| Kappa^f^ | 0.23 | 0.15 |
| *PAU-7S, calibrated* |  |  |
| MVPA, min/d (SD) | 123.0 (15.8) | 68.1 (21.2) |
| Between-method difference, min/d (95% CI)^a^ | 9.9 (5.5;14.3) | -9.7 (-14.1;-5.2) |
| Proportional agreement, %; (95% CI)^b^ | 115 (110;121) | 95 (89;101) |
| Regression coefficient^c^ | -0.342 (-0.432;-0.252) | -0.258 (-0.363;-0.132) |
| Spearman correlation coefficient | 0.26 | 0.38 |
| Intra-class correlation coefficient | 0.26 | 0.40 |
| Absolute agreement, %^d^ | 59.3 | 60.4 |
| Gross misclassification, %^e^ | 3.3 | 3.2 |
| Kappa^f^ | 0.20 | 0.31 |

CI: confidence interval, PAU-7S: Physical Activity Unit 7-item screener, SD: standard deviation.

^a^ Calculated as: MVPA_PAU-7S – MVPA_accelerometer.

^b^ Calculated as: [MVPA_accelerometer/MVPA-PAU-7S] * 100.

^c^ Regression coefficients (β) between mean of the MVPA and mean differences (independent variable) between MVPA obtained by the PAU-7S and accelerometers.

^d^ Correctly classified terciles of MVPA derived by the PAU-7S and accelerometers.

^e^ Opposite terciles of MVPA derived by the PAU-7S and accelerometers.

^f^ Weighted kappa between terciles of MVPA derived by the PAU-7S and accelerometers.
